# Circumventing HSP90 inhibitors *via* apoptosis block

**DOI:** 10.18632/oncoscience.206

**Published:** 2015-08-20

**Authors:** Dean A. Fennell, Sara Busacca

**Affiliations:** Department of Cancer Studies, Cancer Research UK Leicester Centre, University of Leicester, Leicester, UK

**Keywords:** HSP90, apoptosis, resistance

## Genomic instability and resistance

Heat shock protein 90 (HSP90) is a phylogenetically conserved molecular chaperone that plays a crucial role in regulating cancer cell signalling client networks, and has been described as an evolutionary capacitor that buffers genome variation under normal conditions [1]. Consequently HSP90 inhibition is pleiotropic in its targeting, effectively inhibiting critical cancer networks. Personalizing therapy presents a potential challenge, particularly as knowledge of clinically relevant resistance mechanisms are lacking.

HSP90 inhibition induces genomic instability through induction of aneuploidy [2], which itself fuels clonal selection for resistance. However the significance of aneuploidy with respect to HSP90 inhibitor resistance is currently unknown

Clues regarding possible resistance mechanisms from nature reveal point mutations in the N-domain of HSP90 of H.*Fuscoatra* and S.*hygroscopicus* which confer innate resistance to the benzoquinone ansamycin and the polyketide radicicol antibiotics that they produce respectively [3, 4]. However, evidence to support homologous somatic HSP90 mutations in cancer is lacking. A mutation in the ATP-binding site of HSP90 not only would lead to lack of interaction with the drug but would also inactivate HSP90 function, which is incompatible with survival of the cell.

## Client stability and resistance

Specific clients/oncogenic drivers such as EML4-ALK may be promising molecular therapeutic targets for HSP90 inhibition. We have recently shown however that the structural heterogeneity of EML4-ALK variants exhibit differential sensitivity to HSP90 inhibition due to truncation of the TAPE domain at the translocation breakpoint; variants that completely lack a partial TAPE domain are resistant to HSP90 inhibition [5].

## Insights into resistance through the study of cell death mechanisms

**Figure 1:**
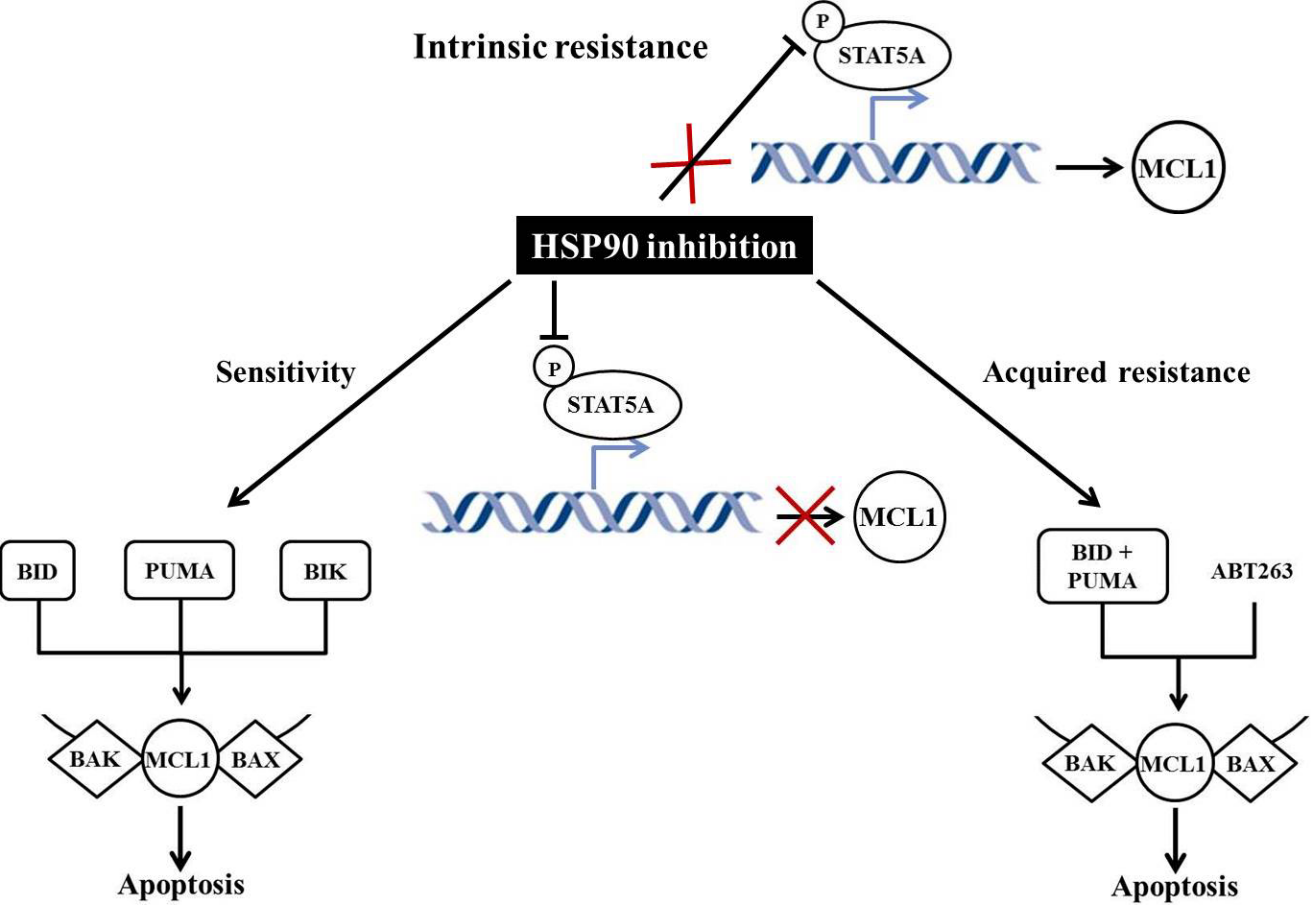
Schematic representation of mechanisms activated following HSP90 inhibition Sensitivity: HSP90 inhibition leads to dephosporylation of STAT5A preventing MCL1 transcription. Sensitive cells are addicted to MCL1 and concurrent downregulation of MCL1 and targeting of BID, BIK and PUMA mediate BAX/BAK-dependent apoptosis. Intrinsic resistance: downregulation of MCL1 following HSP90 inhibition is blocked due to a lack of dephosporylation of STAT5A. Acquired resistance: HSP90 inhibition leads to dephosporylation of STAT5A preventing MCL1 transcription. Resistant cells lose addiction to MCL1 therefore concurrent targeting of BID, PUMA, BCL-xL and BCL-w by ABT737 is required to induce apoptosis.

We recently carried out a functional genetic interrogation to dissect key players involved in the regulation of cell death in response to HSP90 inhibition. Our analysis revealed that HSP90 inhibitors require a complement of multiple BH3s, BIK, BID and PUMA to cooperate in mediating BAX/BAK-dependent mitochondrial apoptosis (Figure 1).

By targeting its client STAT5A, HSP90 also transcriptionally downregulates the anti-apoptotic BCL-2 family member MCL1. Intrinsic resistant cells fail to downregulate MCL1 as the result of a lack of STAT5A dephosphorylation, while conversely, and perhaps surprisingly, cells selected for resistance to HSP90 inhibition, MCL1 repression is conserved (Figure 1), along with other signalling perturbations consistent with on target HSP90 inhibition eg. MAPK and PI3K/AKT/mTOR pathways [6].

We noted a significant correlation between sensitivity to HSP90 inhibition and both downregulation of MCL1 and addiction to MCL1.

In cells addicted to MCL1, its downregulation alone is sufficient to kill by apoptosis and this feature has been demonstrated to correlate with a focal amplification in chromosome 1q21.2 [7], which is one of the most frequent Copy Number Variation (CNV) across human cancers [8]. We show that while de novo, intrinsically resistant cells fail to downregulate MCL1 and are not addicted to MCL1, clones with acquired resistance lose their addiction to MCL1 [6]. These findings therefore suggest that a possible correlation with 1q21 amplification which could be predictive for HSP90 inhibitors granting additional studies.

Sensitivity to HSP90 can be restored through the treatment with a combination of HSP90 inhibitor and the prosurvival BCL-2 family proteins inhibitor ABT737 (which targets BCL-2/xL/w). This effect is not observed in combination whit the BCL-2 selective inhibitor ABT199. This reversion to a sensitive phenotype is effected through BID and PUMA, while BIK becomes redundant (Figure 1). Genome-wide interrogation of CNVs in resistant cells reveals a substantial increase in the mutational burden, but absence of any specific CNVs in either BCL-xL or BCL-w [6].

## Is mitochondrial apoptosis block clinically relevant?

The finding that mitochondrial apoptosis is crucial for HSP90 inhibition efficacy may have significant implications as to predict response and identify patients who will benefit from treatment with HSP90 inhibitors. In the Galaxy 1 trial, the efficacy of the resorcinol 3rd generation HSP90 inhibitor ganetespib in combination with docetaxel was restricted to patients with lung adenocarcinoma having exhibited prior chemosensitivity correlating with treatment greater than 6 months since diagnosis [9], which underpins eligibility of patients enrolling into the follow up Galaxy 2 trial.

In summary, defining the clinical and molecular correlates of resistance to HSP90 inhibitors remains an important challenge in personalizing effective therapy. Exploitation of MCL1 addiction and rationally overcoming acquired resistance may present one strategy for optimizing the use of this exciting drug class.
